# Combination of Glycinamide and Ascorbic Acid Synergistically Promotes Collagen Production and Wound Healing in Human Dermal Fibroblasts

**DOI:** 10.3390/biomedicines10051029

**Published:** 2022-04-29

**Authors:** Ji Eun Lee, Yong Chool Boo

**Affiliations:** 1 Department of Biomedical Science, The Graduate School, Kyungpook National University, 680 Gukchaebosang-ro, Jung-gu, Daegu 41944, Korea; leeje0825@naver.com; 2 BK21 Plus KNU Biomedical Convergence Program, Kyungpook National University, 680 Gukchaebosang-ro, Jung-gu, Daegu 41944, Korea; 3 Department of Molecular Medicine, School of Medicine, Kyungpook National University, 680 Gukchaebosang-ro, Jung-gu, Daegu 41944, Korea; 4 Cell and Matrix Research Institute, Kyungpook National University, 680 Gukchaebosang-ro, Jung-gu, Daegu 41944, Korea

**Keywords:** dermal fibroblasts, collagen production, glycinamide, glycine, ascorbic acid, wound healing

## Abstract

The purpose of this study is to present a novel strategy to enhance collagen production in cells. To identify amino acid analogs with excellent collagen production-enhancing effects, human dermal fibroblasts (HDFs) were treated with 20 kinds of amidated amino acids and 20 kinds of free amino acids, individually at 1 mM. The results showed that glycinamide enhanced collagen production (secreted collagen level) most effectively. Glycine also enhanced collagen production to a lesser degree. However, other glycine derivatives, such as N-acetyl glycine, N-acetyl glycinamide, glycine methyl ester, glycine ethyl ester, and glycyl glycine, did not show such effects. Glycinamide increased type I and III collagen protein levels without affecting *COL1A1* and *COL3A1* mRNA levels, whereas transforming growth factor-β1 (TGF-β1, 10 ng mL^−1^) increased both mRNA and protein levels of collagens. Ascorbic acid (AA, 1 mM) increased *COL1A1* and *COL3A1* mRNA and collagen I protein levels. Unlike TGF-β1, AA and glycinamide did not increase the protein level of α-smooth muscle actin, a marker of differentiation of fibroblasts into myofibroblasts. The combination of AA and glycinamide synergistically enhanced collagen production and wound closure in HDFs to a level similar to that in cells treated with TGF-β1. AA derivatives, such as magnesium ascorbyl 3-phosphate (MAP), 3-*O*-ethyl ascorbic acid, ascorbyl 2-*O*-glucoside, and ascorbyl tetraisopalmitate, enhanced collagen production, and the mRNA and protein levels of collagens at 1 mM, and their effects were further enhanced when co-treated with glycinamide. Among AA derivatives, MAP had a similar effect to AA in enhancing wound closure, and its effect was further enhanced by glycinamide. Other AA derivatives had different effects on wound closure. This study provides a new strategy to enhance cell collagen production and wound healing using glycinamide in combination with AA.

## 1. Introduction

Collagen is the most abundant protein in animals and accounts for three-quarters of the dry skin mass. Collagen exists in the form of a triple helix, and its primary amino acid sequence repeats glycine-proline-X or glycine-X-hydroxyproline, where X may be any amino acid other than glycine, proline, or hydroxyproline [1]. To date, as many as 28 different types of collagen have been found [2], and the most common type is collagen I, which accounts for more than 90% of human collagen [3]. Normal human skin contains various types of collagen, i.e., 80–90% collagen I, 8–12% collagen III, and 5% collagen V [4]. As a main component of the extracellular matrix (ECM) in the dermis, collagen provides structural support and maintains the firmness and elasticity of the skin [5].

Insufficient or excessive levels of dermal collagen are associated with various skin diseases and aesthetic problems. Collagen disease can occur when there is a defect in collagen production and maturation due to genetic causes, when collagen production is impaired due to a lack of ascorbic acid (AA), and when collagen production is excessively increased due to an autoimmune reaction [6].

The skin has evolved efficient mechanisms to heal wounds involving the following four main phases: hemostasis, inflammation, proliferation, and remodeling of ECM [7]. Fibroblasts are the major cell type responsible for wound ECM remodeling through the production of collagen and other components [8]. Collagen interacts with cells and regulates the wound healing process [9].

Alteration in the amount and structure of collagen is a common feature of natural aging and photoaging of the skin [10]. In aging skin, matrix metalloproteinases that degrade collagen are increased while the transforming growth factor (TGF)-β1 that stimulates collagen production is reduced [11,12]. As the skin ages, the ratio of collagen III to collagen I decreases, and skin tension, elasticity, and healing tend to decrease [13]. Thus, controlling the production of collagen is important not only from a medical point of view but also from a cosmetic point of view.

The production and secretion of collagen proceeds through several complex steps [14]. TGF-β1 is an important member of the TGF-β family that promotes the proliferation, collagen formation, and differentiation of the cells [15]. AA can increase collagen production via multiple mechanisms [16,17]. As an essential cofactor of prolyl hydroxylase and lysyl hydroxylase, AA modulates collagen production at the post-translational steps [18,19]. AA is also known to stimulate procollagen I and III gene transcription in human dermal fibroblasts (HDFs) [20,21].

Since ECM proteins, including collagen, have a unique amino acid composition, their production in dermal fibroblasts is affected by the availability of certain amino acids [22,23,24,25]. Glutamine or glutamate increases collagen production by promoting proline production via pyrroline 5-carboxylate [26]. The external supply of proline increases collagen production, and the effect was greater in the glutamine-deficient medium [27]. Paz-Lugo et al. compared the effects of various amino acids on collagen production in articular chondrocytes and found that glycine had a stronger effect on collagen production than proline, lysine, and other amino acids [28].

The purpose of this study is to find an optimized condition for enhancing collagen production. We first compared 20 kinds of amidated amino acids on collagen production in HDFs. Since glycinamide most effectively promoted collagen production, we proceeded with further research focusing on this compound. The effect of glycinamide was additionally compared with that of 20 kinds of free amino acids and various glycine derivatives. Its effects and mechanisms of action were compared with those of AA. The effects of the combination of AA and glycinamide on collagen production and wound closure were investigated. Furthermore, the effects of the combination between glycinamide and several types of AA derivatives were explored. This study provided a new strategy to effectively enhance cell collagen production and wound healing at the cell level.

## 2. Materials and Methods

### 2.1. Reagents

Twenty kinds of free amino acids and twenty kinds of amidated amino acids were used in this study. The compounds are listed in Table 1 using the single-letter codes for amino acids. The free amino acids were purchased from Sigma-Aldrich (St. Louis, MO, USA). The amidated amino acids were purchased from Watanabe Chemical Ind., Ltd. (Hiroshima, Japan). N-Acetyl glycine, N-acetyl glycinamide, glycine methyl ester HCl, glycine ethyl ester HCl, glycyl glycine, AA, 3-*O*-ethyl ascorbic acid (EAA), and ascorbyl 2-*O*-glucoside (AG) were purchased from Sigma-Aldrich. Magnesium ascorbyl 3-phosphate (MAP) and ascorbyl tetraisopalmitate (ATI) were purchased from Biosynth Carbosynth (Berkshire, UK). TGF-β1 was purchased from R&D Systems (Minneapolis, MN, USA).

### 2.2. Cell Culture

HDFs derived from adult skin were obtained from Cascade Biologics (Portland, OR, USA). Cells were cultured in a closed incubator at 37 °C in a humidified atmosphere containing 5% CO_2_. The growth medium was Iscove’s modified Dulbecco’s medium (IMDM) supplemented with 10% fetal bovine serum (Gibco BRL, Grand Island, NY, USA) and 1% antibiotics (100 U mL^−1^ penicillin, 0.1 mg mL^−1^ streptomycin, and 0.25 µg mL^−1^ amphotericin B) (Thermo Fisher, Waltham, MA, USA).

### 2.3. Cell Viability Assay

The viability of cells was estimated using 3-(4,5-dimethylthiazol-2-yl)-2,5-diphenyltetrazolium bromide (MTT) [29]. Briefly, HDFs were cultured in the growth medium in 96-well culture plates (1 × 10^4^ cells per well) for 24 h and then treated with each drug for 48 h. After discarding the medium, cells were incubated in a fresh growth medium (100 µL) containing 1 mg mL^−1^ MTT (Amresco, Solon, OH, USA) for 2 h at 37 °C. The medium was eliminated by aspiration, and the formazan dye accumulated inside the cells was dissolved in 100 µL dimethyl sulfoxide. The absorbance of the solution was measured at 570 nm using a Spctrostar Nano microplate reader (BMG LABTECH GmbH, Ortenberg, Germany).

### 2.4. Measurement of Collagen Secreted

The collagen protein levels of the conditioned medium were assessed using the Sircol™ Collagen Assay (Biocolor, Antrim, UK) according to the manufacturer’s instructions. HDFs were cultured in the growth medium in 12-well plates (2 × 10^5^ cells per well) for 24 h, followed by the treatment with each drug for an additional 24 h. After discarding the medium and washing the cells with 500 µL phosphate-buffered saline (PBS), cells were incubated in 1 mL serum-free IMDM for an additional 24 h. The 1.0 mL medium sample (the conditioned medium, or fresh medium as a negative control) was transferred to a microcentrifuge tube, mixed with 100 µL collagen isolation & concentration reagent, and incubated overnight at 4 °C. The samples were centrifuged with an Eppendorf centrifuge 5418R (Eppendorf, Barkhausenweg, Hamburg, Germany) at 14,500× *g* for 10 min at 4 °C and the upper layer was carefully removed using a pipette without disturbing the lower layer. After each tube was added, 500 µL Sircol dye reagent and the samples were inverted 5 times and shaken for 30 min at 25 °C. After centrifugation, the supernatants were discarded and the pellets were washed with 500 µL ice-cold acid-salt wash reagent followed by an additional centrifugation step. Then, the pellets were dissolved in 250 µL alkali reagent and 200 µL portions were transferred to a 96-well plate. The optical density of the solution was read at 555 nm using a Spectrostar Nano microplate reader.

### 2.5. Quantitative Reverse Transcriptase-Polymerase Chain Reaction (qRT-PCR) Analysis

HDFs were cultured at 2 × 10^5^ cells per well on a 6-well plate and treated with each drug for 12 h or 24 h. Total RNA was isolated using an RNeasy kit (Qiagen, Valencia, CA, USA) and the complementary DNA (cDNA) was generated from 1 μg of total RNA by reverse transcription using the High-Capacity cDNA Archive Kit (Applied Biosystems, Foster City, CA, USA). The qRT-PCR was performed using a StepOnePlus™ Real-Time PCR System (Applied Biosystems). Briefly, the reaction mixture (20 µL) consisted of SYBR^®^ Green PCR Master Mix (Applied Biosystems), 60 ng cDNA, and 2 pmol gene-specific primer sets. The cycling parameters were set as follows: 50 °C for 2 min, 95 °C for 10 min, 40 amplification cycles of 95 °C for 15 s, and 60 °C for 1 min. In each PCR run, the homogeneity of the PCR product was confirmed by the melting curve analysis. The mRNA levels of collagen I alpha 1 chain (*COL1A1*) and collagen III alpha 1 chain (*COL3A1*) were compared with that of glyceraldehyde 3-phosphate dehydrogenase (*GAPDH*), a housekeeping reference gene, using the comparative C_t_ method. C_t_ is defined as the number of cycles required for the PCR signal to exceed the threshold. Fold changes in the test group compared to the control group were calculated as 2^−∆∆^^Ct^, where ∆∆C_t_ = ∆C_t__(test)_ − ∆C_t__(control)_ = [C_t__(gene, test)_ − C_t__(reference, test)_] − [C_t__(gene, control)_ − C_t__(reference, control)_].

The gene-specific primers (2 pmol) were purchased from Macrogen (Seoul, Korea) and their sequences are shown in Table 2.

### 2.6. Western Blot Analysis

Primary antibodies for collagen I (#293182), collagen III (#514601), and β-actin (#47778) were purchased from Santa Cruz Biotechnology (Santa Cruz, CA, USA) and α-smooth muscle action (#2547) was purchased from Sigma-Aldrich. Anti-mouse IgG (#7076) secondary antibody conjugated to horseradish peroxidase was purchased from Cell Signaling Technology (Danvers, MA, USA). Sodium dodecyl sulfate (SDS)-polyacrylamide gel electrophoresis (PAGE) and Western blotting were performed as previously described [32]. A portion of cell lysate (20 µg protein) was mixed with Laemmli sample buffer (5×) and heated at 95 °C for 5 min to denature the protein. Proteins were resolved in 7.5% (*w*/*v*) SDS-polyacrylamide gels at 100 V and electrically transferred to a polyvinylidene difluoride membrane (Amersham Pharmacia, Little Chalfont, UK) at 100 V for 1 h, 80 V for 1 h and 40 V overnight at 4 °C. After blocking incubation with the antibody dilution buffer (137 mM NaCl, 20 mM Tris-Cl, 0.1% Tween 20, 5% skim milk, pH 7.6.), the membrane was incubated with a primary antibody solution at 4 °C overnight, followed by incubation with a secondary antibody solution at 25 °C for 1 h. Antibody dilution ratios are as follows: collagen I, collagen III, and β-actin antibodies, 1:1000; α-smooth muscle actin antibody, 1:20,000; the secondary antibody, 1:5000). The target protein bands on the membrane were visualized by a chemiluminescence method using the picoEPD Western Reagent kit (ELPIS-Biotech, Daejeon, Korea). Image J program from U. S. National Institutes of Health (Bethesda, MD, USA) was used in analyzing the captured blot images.

### 2.7. Wound Healing Assay

Wound healing assay was performed by using Oris™ Universal Cell Migration Assembly kit (Platypus Technologies, WI, USA) according to the manufacturer’s instructions. After Oris™ stoppers were inserted into each well of the Oris™-compatible 96-well plate, the plate was turned over and the complete attachment of stoppers was confirmed. The suspension of HDFs in IMDM was added to each well (1 × 10^4^ cells in 100 µL per well) and cells were incubated for 12 h to permit cell attachment. The stoppers were then removed using the stopper tool, and the medium was removed using the pipette. After washing gently with 100 µL PBS to eliminate any unattached cells, the adherent cells were treated with each drug in a 100 µL culture medium for the specified time. Images of the wounds were obtained using a phase-contrast microscope (Eclipse TS100, Nikon Instruments Inc., Melville, NY, USA). Wound areas in the images were quantitated using the NIH Image J program. Wound closure (%) was calculated using the following equation: Wound closure (%) = [(A_0_ − A_t_)/A_0_] × 100, where A_0_ is the area of the original wound and A_t_ is the area of wound measured at a specified time [33].

### 2.8. Statistical Analysis

The experimental results are presented as the mean ± standard deviation (SD) of three or more independent experiments. SigmaStat v.3.11 Statistical Analysis Software (Systat Software Inc, San Jose, CA, USA) was used in the statistical analysis of data. A one-way analysis of variance (ANOVA) at *p* < 0.05 level was performed to determine the existence of different group means. All experimental groups were compared to each other using Duncan’s multiple range tests.

## 3. Results

### 3.1. Effects of Various Amidated and Free Amino Acids on the Collagen Production of HDFs

**Figure 1:**
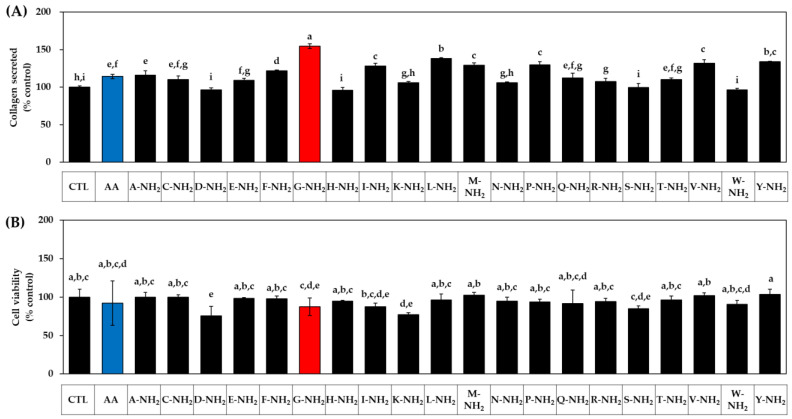
Effects of twenty amidated amino acids on the collagen production and the viability of human dermal fibroblasts (HDFs). Ascorbic acid (AA) was used as a positive control. Cells were treated with vehicle (CTL), AA, or each amidated amino acid at 1 mM. After 48 h, the collagen protein secreted into the conditioned medium was quantitated (**A**). The viability of the adherent cells was determined (**B**). Data are presented as % of control (means ± SD, *n* = 3 for (**A**), and *n* = 4 for (**B**)). Duncan’s multiple range test was performed to compare all group means to each other. Groups that share the same alphabet letters (a–i) do not have significantly different means at the *p* < 0.05 level.

In the first experiment, we compared all 20 kinds of C-terminal amidated amino acids with AA. The HDFs were treated with each amidated amino acid or AA at 1 mM. After 48 h, the conditioned medium was harvested and used for the quantitation of collagen protein by a colorimetric method. The adherent cells were used for the assessment of cell viability using MTT. As shown in Figure 1A, many kinds of amidated amino acids enhanced the collagen production (secreted collagen level), glycinamide(G-NH_2_) being the most effective, followed by leucinamide (L-NH_2_), tyrosinamide (Y-NH_2_), valinamde (V-NH_2_), prolinamide (P-NH_2_), methioninamide (M-NH_2_), isoleucinamide (I-NH_2_), phenylalaninamide (F-NH_2_), alaninamide (A-NH_2_), glutaminamide (Q-NH_2_), cysteinamide (C-NH_2_), threoninamide (T-NH_2_), glutamic acid α-amide (E-NH_2_), and argininamde (R-NH_2_). However, other amidated amino acids, such as asparaginamide (N-NH_2_), lysinamide (C-NH_2_), serinamide (S-NH_2_), tryptophanamide (W-NH_2_), aspartic acid α-amide (D-NH_2_), and histidinamide (H-NH_2_) had no significant effects. Ascorbic acid, used as a positive control, also significantly increased the collagen production, but the change was smaller than that for glycinamide. As for the effect on cell viability, lysinamide and aspartic acid α-amide had decreasing effects, and other amidated amino acids had no significant effects, as shown in Figure 1B. Therefore, glycinamide was identified to most effectively enhance cellular collagen production.

**Figure 2:**
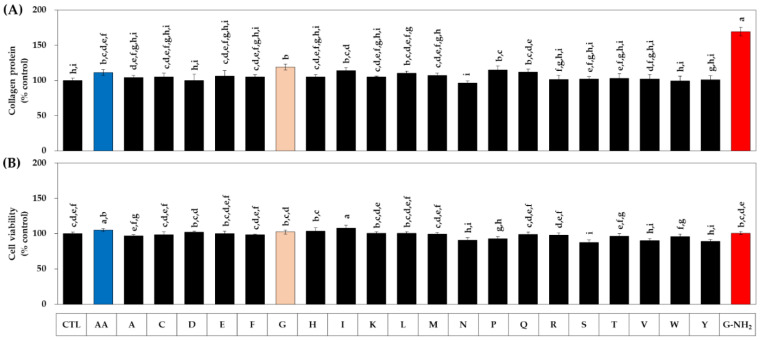
Effects of twenty free amino acids on the collagen production and the viability of HDFs. AA was used as a positive control. Cells were treated with vehicle (CTL), AA, or each amino acid at 1 mM. After 48 h, the collagen protein secreted into the conditioned medium was quantitated (**A**). The viability of the adherent cells was determined (**B**). Data are presented as % of control (means ± SD, *n* = 3 for (**A**), and *n* = 4 for (**B**)). Groups that share the same alphabet letters (a–i) do not have significantly different means at the *p* < 0.05 level.

In the next experiment, we compared 20 kinds of naturally occurring free amino acids with AA. HDFs were treated with each amino acid or AA at 1 mM for 48 h. As shown in Figure 2A, among the free amino acids, glycine (G) increased collagen most effectively, followed by proline (P), isoleucine (I), glutamine (Q), and leucine (L), and other amino acids had no effect. In this experiment, glycinamide was also compared, and it was confirmed that its collagen-promoting effect was superior to all free amino acids. As for the effect on cell viability, several kinds of amino acids had a significant effect on cell viability to a small extent (Figure 2B).

### 3.2. Effects of Glycine Analogs on the Collagen Production of HDFs

**Figure 3:**
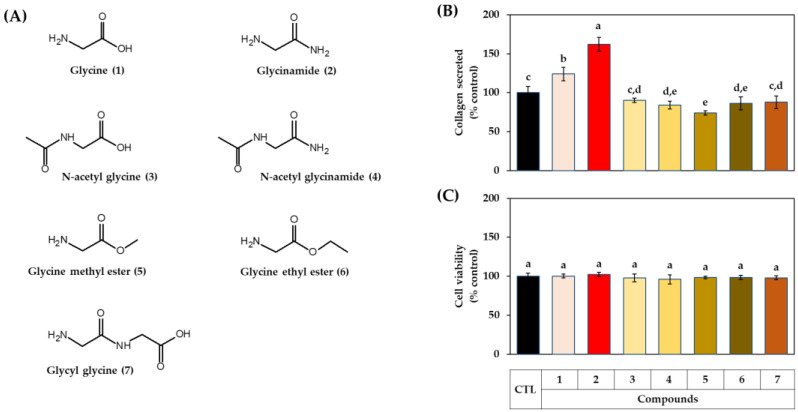
Effects of glycine analogs on collagen production and the viability of HDFs. Chemical structures of glycine (1), glycinamide (2), N-acetyl glycine (3), N-acetyl glycinamide (4), glycine methyl ester (5), glycine ethyl ester (6), and glycyl glycine (7) are shown (**A**). Cells were treated with vehicle (CTL) or each compound at 1 mM. After 48 h, the collagen protein secreted into the conditioned medium was quantitated (**B**). The viability of the adherent cells was determined (**C**). Data are presented as % of control (means ± SD, *n* = 3 for (**A**), and *n* = 4 for (**B**)). Groups that share the same alphabet letters (a–e) do not have significantly different means at the *p* < 0.05 level.

In the following experiment, the effects of various glycine analogs (Figure 3A) on cell collagen production were compared. When the HDFs were treated with each compound at 1 mM, collagen production was highly increased in the order of glycinamide and glycine (Figure 3B). The other glycine analogs, such as N-acetyl glycine, glycine ethyl ester, and glycyl glycine, did not affect collagen production, whereas N-acetyl glycinamide and glycine methyl ester had a rather decreasing effect. All glycine analogs had no significant effect on cell viability (Figure 3C). The results suggest that glycinamide may be an optimized glycine analog for enhancing cellular collagen production.

### 3.3. Dose-dependent Effects of AA, Glycine, and Glycinamide on the Collagen Production of HDFs

**Figure 4:**
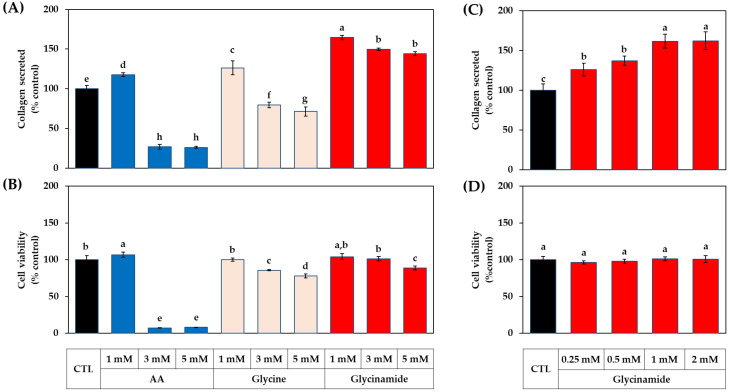
Dose-dependent effects of AA, glycine, and glycinamide on the collagen production of HDFs. Cells were treated with vehicle (CTL) or 1–5 mM of each compound (**A**,**B**) or 0.25–2 mM of glycinamide (**C**,**D**). After 48 h, the collagen protein secreted into the conditioned medium was quantitated (**A**,**C**). The viability of the adherent cells was determined (**B**,**D**). Data are presented as % of control (means ± SD, *n* = 3 for (**A**–**C**), and *n* = 4 for (**D**)). Groups that share the same alphabet letters (a–h) do not have significantly different means at the *p* < 0.05 level.

The next experiment compared the collagen production-enhancing effects depending on the concentrations of AA, glycine, and glycinamide. As shown in Figure 4A,B, AA increased collagen production at 1 mM without affecting cell viability but significantly reduced cell viability at 3–5 mM. Glycine at 1 mM increased collagen production without affecting cell viability, but at 3–5 mM, it significantly reduced cell viability. Glycinamide at 1 mM promoted collagen production without affecting cell viability, but at 3–5 mM, it slightly decreased cell viability. As shown in Figure 4C,D, glycinamide enhanced collagen production even at a low concentration of 0.25 mM and showed the greatest effect at 1–2 mM without decreasing cell viability. These results suggest that the optimal concentration of glycinamide to promote collagen production is in the range of 1–2 mM.

### 3.4. Effects of AA, Glycinamide, and TGF-β1 on the Collagen Production of HDFs

**Figure 5:**
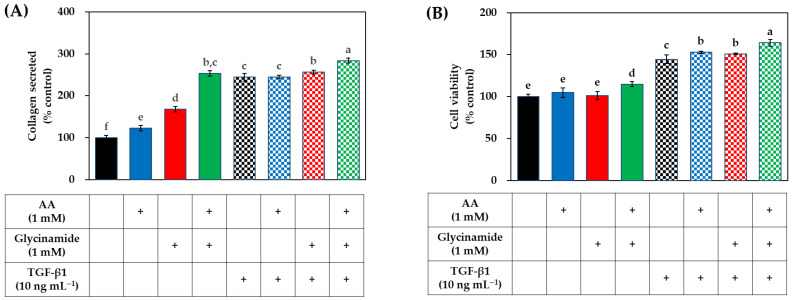
Effects of individual and combined treatments of AA, glycinamide, and transforming growth factor (TGF)-β1 on the collagen production in HDFs. Cells were treated with AA, glycinamide, or TGF-β1 each or in combinations. After 48 h, the collagen protein secreted into the conditioned medium (**A**) and the viability of the adherent cells (**B**) were determined. Data are presented as % of control (means ± SD, *n* = 3 for (**A**), and *n* = 5 for (**B**)). Groups that share the same alphabet letters (a–f) do not have significantly different means at the *p* < 0.05 level.

We further investigated the effect of combined treatment with AA and glycinamide on collagen production and cell viability in the absence or presence of TGF-β1. As shown in Figure 5A, in the absence of TGF-β1, AA (1 mM) and glycinamide (1 mM) increased collagen production by 23.1% and 68.2%, respectively, and their combination increased collagen production by 153%, which is much bigger than the arithmetic sum of the effects of each treatment (91.3%). These results suggest that the combination of AA and glycinamide can synergistically promote collagen production. The increase in collagen production by the combination of AA and glycinamide was similar to that by TGF-β1 (10 ng mL^−1^). The collagen production-enhancing effect of a combination of AA and glycinamide was also observed in the presence of TGF-β1.

As shown in Figure 5B, TGF-β1 significantly increased viable cells compared to the control group by 44.5%. When AA and glycinamide were treated individually, there was no significant change in cell viability, but when they were treated in combination, viable cells were increased compared to the control group by 14.8%. This result suggests that the combination of AA and glycinamide may synergistically promote the proliferation of HDFs.

### 3.5. Effects of AA, Glycinamide, and TGF-β1 on the mRNA and Protein Levels of Collagens in HDFs

**Figure 6:**
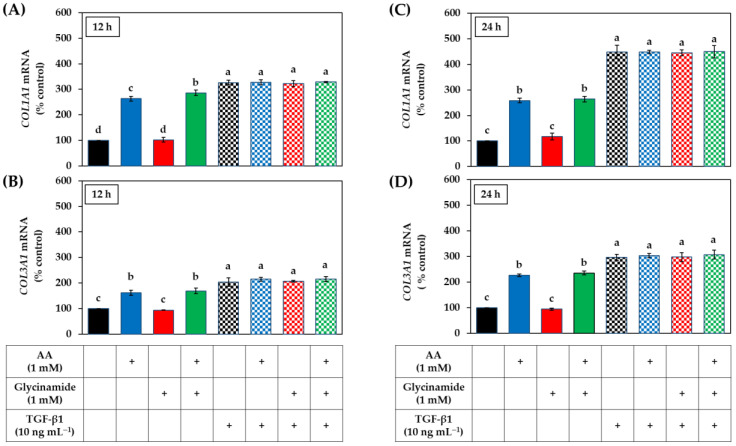
Effects of individual and combined treatments of AA, glycinamide, and TGF-β1 on the mRNA expression of procollagen genes in HDFs. Cells were treated with AA, glycinamide, or TGF-β1 each or in combinations for 12 h (**A**,**B**) or 24 h (**C**,**D**). The mRNA levels of *COL1A1* (**A**,**C**) and *COL3A1* (**B**,**D**) were analyzed by quantitative reverse transcriptase-polymerase chain reaction (qRT-PCR) and the results were normalized to that of glyceraldehyde 3-phosphate dehydrogenase (*GAPDH*). Data are presented as % of control (means ± SD, *n* = 3). Groups that share the same alphabet letters (a–d) do not have significantly different means at the *p* < 0.05 level.

We hypothesized that the synergistic effect of AA and glycinamide might be due to their actions at different stages of collagen production. To verify this hypothesis, the mRNA levels of the procollagen genes *COL1A1* and *COL3A1* were analyzed by qRT-PCR after 12 h and 24 h after HDFs were treated with AA, glycinamide, and TGF-β1 individually or in combination. As shown in Figure 6, in the absence of TGF-β1, AA significantly increased the mRNA levels of *COL1A1* and *COL3A1* at both time points, whereas glycinamide had no such effect. When AA and glycinamide were treated in combination, the effect was similar to that of AA alone. TGF-β1 alone also increased the mRNA levels of *COL1A1* and *COL3A1*, as expected. In the presence of TGF-β1, no further increase in mRNA levels of *COL1A1* and *COL3A1* by AA or glycinamide was observed.

**Figure 7:**
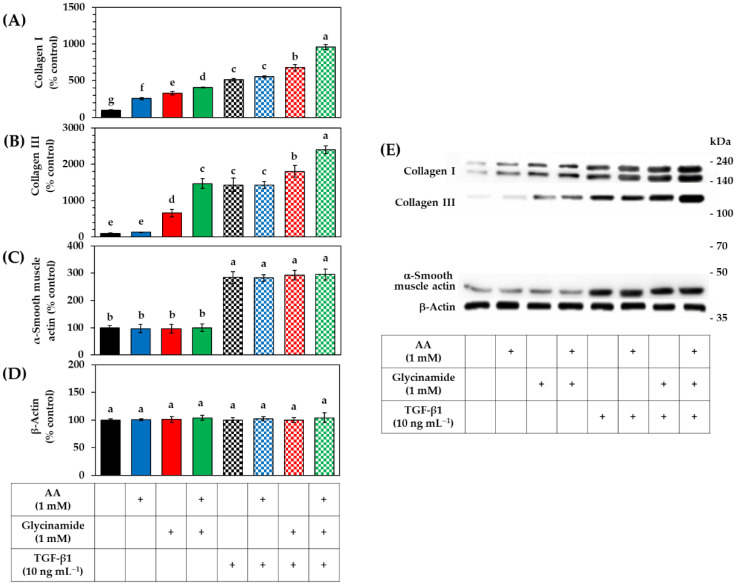
Effects of individual and combined treatments of AA, glycinamide, and TGF-β1 on the protein levels of collagen I, III, α-smooth muscle actin, and β-actin in HDFs. Cells were treated with AA, glycinamide, or TGF-β1 each or in combinations for 24 h. The intracellular protein levels of collagen I (**A**), collagen III (**B**), α-smooth muscle actin (**C**), and β-actin (**D**) were analyzed by Western blot. A reconstituted image of typical blots is shown with molecular weight markers (**E**). Data are presented as % of control (means ± SD, *n* = 3). Groups that share the same alphabet letters (a–g) do not have significantly different means at the *p* < 0.05 level.

The effects of AA, glycinamide, TGF-β1, and their combinations on the collagen protein levels were analyzed by Western blot. As shown in Figure 7A, AA and glycinamide increased collagen I level. When cells were treated with them in combination, the collagen I level was further increased. As shown in Figure 7B, AA had no significant effect on the collagen III level, whereas glycinamide increased it significantly. When cells were treated with AA plus glycinamide, collagen III level was further increased. TGF-β1 increased collagen I and III levels, and this effect was further enhanced by glycinamide alone, or glycinamide plus AA, but not by AA alone. As shown in Figure 7C, TGF-β1 increased the level of α-smooth muscle actin, indicating the differentiation of the dermal fibroblasts into myofibroblasts [34]. However, AA or glycinamide did not show such effects. As shown in Figure 7D, the level of β-actin, which was used as a control, did not change with any treatments.

Collectively, these results suggest that AA acts at the transcriptional stage by stimulating the mRNA expression of procollagen genes and that glycinamide acts at the translational stage by providing the building blocks required for the synthesis of collagen proteins. It is also suggested that AA and glycinamide enhance collagen production without inducing the differentiation of dermal fibroblasts into myofibroblasts.

### 3.6. Effects of AA, Glycinamide, and TGF-β1 on the Wound Closure in HDFs

**Figure 8:**
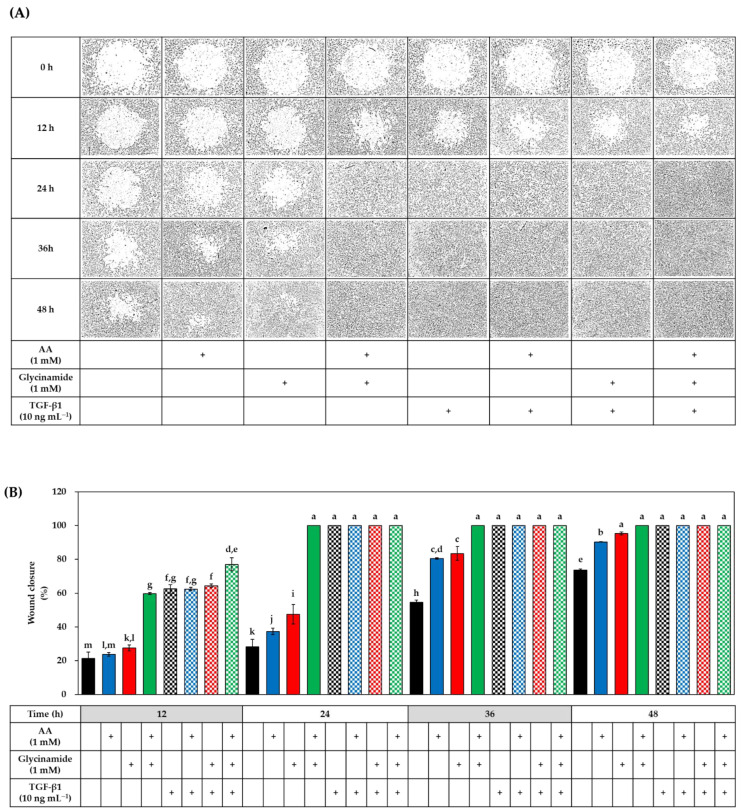
Effects of individual and combined treatments of AA, glycinamide, and TGF-β1 on the wound closure in HDFs. Cells were cultured for 12 h in the presence of a stopper to create a small circular wound. After removing the stopper, cells were treated with AA, glycinamide, or TGF-β1 each or in combinations for 48 h. Representative microscopic wound images before and after treatments are shown in panel (**A**). Quantitated data for wound closure (%) at different time points are shown in panel (**B**) (means ± SD, *n* = 3). Groups that share the same alphabet letters (a–m) do not have significantly different means at the *p* < 0.05 level.

In the next experiment, we examined whether AA, glycinamide, and TGF-β1 alone or in combination could promote wound healing in HDFs. A circular wound was created by culturing cells in the wells attached by a stopper. After removing the stopper, cells were treated with either vehicle or drugs for 48 h. Representative microscopic wound images before and after treatments are shown in Figure 8A. At the baseline, all groups had the same degree of the wound, and the wound was gradually closed over time in the control and test groups. Relative wound closure was quantitated via image analysis, and data are shown in Figure 8B. In the absence of TGF-β1, at 12 h after treatments, AA and glycinamide each significantly enhanced wound closure compared to the control group, and when cells were treated with them in combination, wound closure was further enhanced, reaching a level similar to that of the TGF-β1 group. The change by the combined treatment of AA and glycinamide (32.1%) was much bigger than the arithmetic sum of the changes by individual treatment of AA and glycinamide (2.5% + 6.3% = 8.8%). These results suggest that the combination of AA and glycinamide can synergistically promote the wound closure. The synergy effects of the combined treatment of AA and glycinamide were also observed at 24 h after treatment. Even in the presence of TGF-β1, co-treatment of AA and glycinamide enhanced the wound healing further.

### 3.7. Effects of Various AA Analogs, Glycinamide, and TGF-β1 on the Collagen Production of HDFs

**Figure 9:**
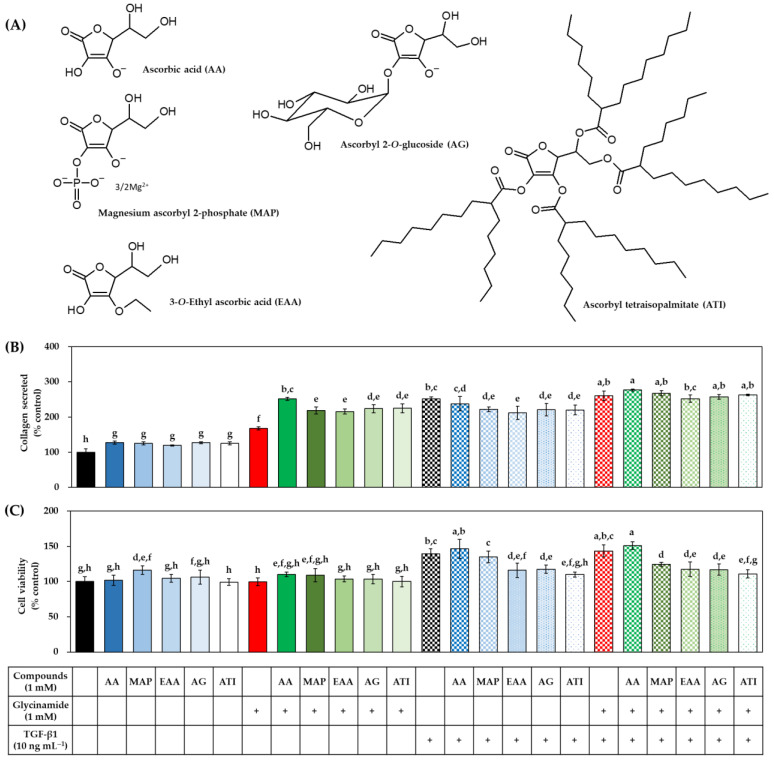
Effects of individual and combined treatments of AA analogs, glycinamide, and TGF-β1 on the collagen production in HDFs. Chemical structures of AA analogs, such as AA, magnesium ascorbyl 3-phosphate (MAP), 3-*O*-ethyl ascorbic acid (EAA), ascorbyl 2-*O*-glucoside (AG), and ascorbyl tetraisopalmitate (ATI), are shown (**A**). Cells were treated with an AA analog alone or in combination with glycinamide and/or TGF-β1. After 48 h, the collagen protein secreted into the conditioned medium (**B**) and the viability of the adherent cells (**C**) were measured. Data are presented as % of control (means ± SD, *n* = 3 for (**A**), and *n* = 5 for (**B**)). Groups that share the same alphabet letters (a–h) do not have significantly different means at the *p* < 0.05 level.

To investigate the effect of various AA analogs (Figure 9A) on collagen production, the HDFs were treated with each analog alone or in combination with glycinamide and/or TGF-β1, and the collagen production and cell viability were evaluated. AA derivatives, such as MAP, EAA, AG, and ATI, significantly enhanced the collagen production to a degree similar to that of AA (Figure 9B). When the cells were treated with each AA analog in combination with glycinamide, collagen production was further enhanced. The changes by the combined treatment of glycinamide and each AA analog (MAP, 118.4%; EAA, 115.5%; AG, 123.6%; ATI, 124.6%) were bigger than the arithmetic sums of the changes by individual treatment of glycinamide and each AA analog (MAP, 25.3% + 67.4% = 92.7%; EAA, 19.5% + 67.4% = 86.9%; AG, 27.2% + 67.4% = 94.6%; ATI, 25.5% + 67.4% = 92.9%). These results suggest that the combination of AA analogs and glycinamide can synergistically promote collagen production. The viability of cells was not significantly affected when treated with each AA analog alone or in combination with glycinamide (Figure 9C). However, some AA derivatives (EAA, EG, and ATI) significantly reduced the cell proliferation effect of TGF-β1.

### 3.8. Effects of Various AA Analogs, Glycinamide, and TGF-β1 on the mRNA and Protein Levels of Collagens in HDFs

**Figure 10:**
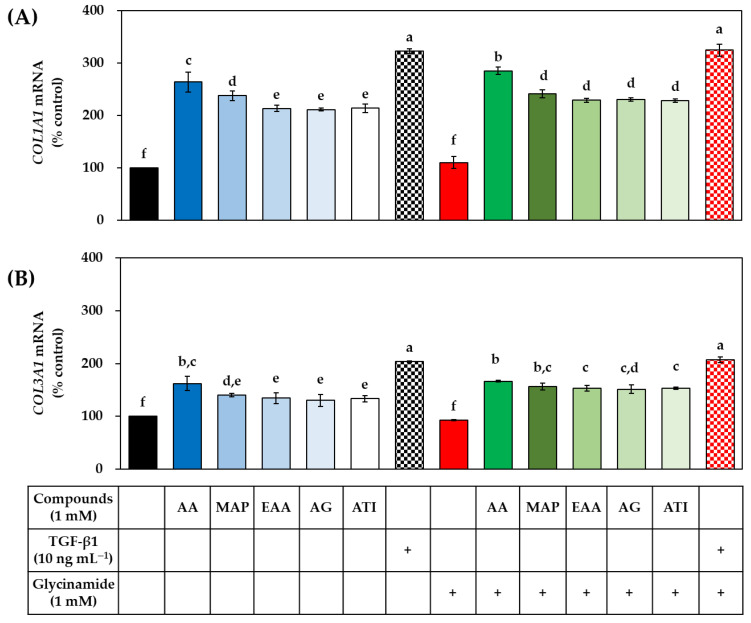
Effects of individual and combined treatments of AA analogs (such as AA, MAP, EAA, AG, and ATI), glycinamide, and TGF-β1 on the mRNA expression of procollagen genes in HDFs. Cells were treated with an AA analog or TGF-β1 in the absence or presence of glycinamide for 12 h. The mRNA levels of *COL1A1* (**A**) and *COL3A1* (**B**) were analyzed by qRT-PCR and the results were normalized to that of *GAPDH*. Data are presented as % of control (means ± SD, *n* = 3). Groups that share the same alphabet letters (a–f) do not have significantly different means at the *p* < 0.05 level.

The effects of several AA analogs on the mRNA and protein levels of collagens were compared in the absence or presence of glycinamide. TGF-β1 was tested for comparative purposes. As shown in Figure 10, all the AA analogs tested and TGF-β1 increased the mRNA levels of *COL1A1* and *COL3A1*, and these effects were not at all or very little altered by glycinamide.

**Figure 11:**
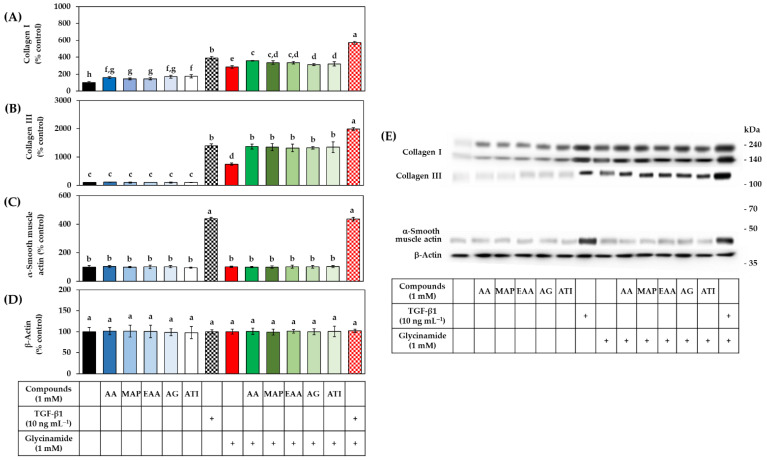
Effects of individual and combined treatments of AA analogs (such as AA, MAP, EAA, AG, and ATI), glycinamide, and TGF-β1 on the protein levels of collagen I, III, α-smooth muscle actin, and β-actin in HDFs. Cells were treated with an AA analog or TGF-β1 in the absence or presence of glycinamide for 24 h. The intracellular levels of collagen I (**A**), collagen III (**B**), α-smooth muscle actin (**C**), and β-actin (**D**) were analyzed by Western blot. A reconstituted image of typical blots is shown with molecular weight markers (**E**). Data are presented as % of control (means ± SD, *n* = 3). Groups that share the same alphabet letters (a–h) do not have significantly different means at the *p* < 0.05 level.

As shown in Figure 11A, the AA analogs (AA, MAP, EAA, AG, and ATI) and glycinamide increased the level of collagen I, and their combinations further enhanced it. As shown in Figure 11B, the AA analogs did not affect the level of collagen III, whereas glycinamide increased it significantly. When the cells were treated with an AA analog plus glycinamide, the level of collagen III was further increased. TGF-β1 increased the levels of collagen I and III, and this effect was further enhanced by glycinamide. As shown in Figure 11C, TGF-β1 increased the level of α-smooth muscle actin, but AA analogs and glycinamide did not show such effects. As shown in Figure 11D, the level of β-actin, which was used as a control, did not change significantly by any treatments. Collectively, these results suggest that glycinamide can enhance collagen I and III levels in the cells stimulated by various AA analogs without affecting cell differentiation and gene transcription.

### 3.9. Effects of Various AA Analogs, Glycinamide, and TGF-β1 on the Wound Closure in HDFs

**Figure 12:**
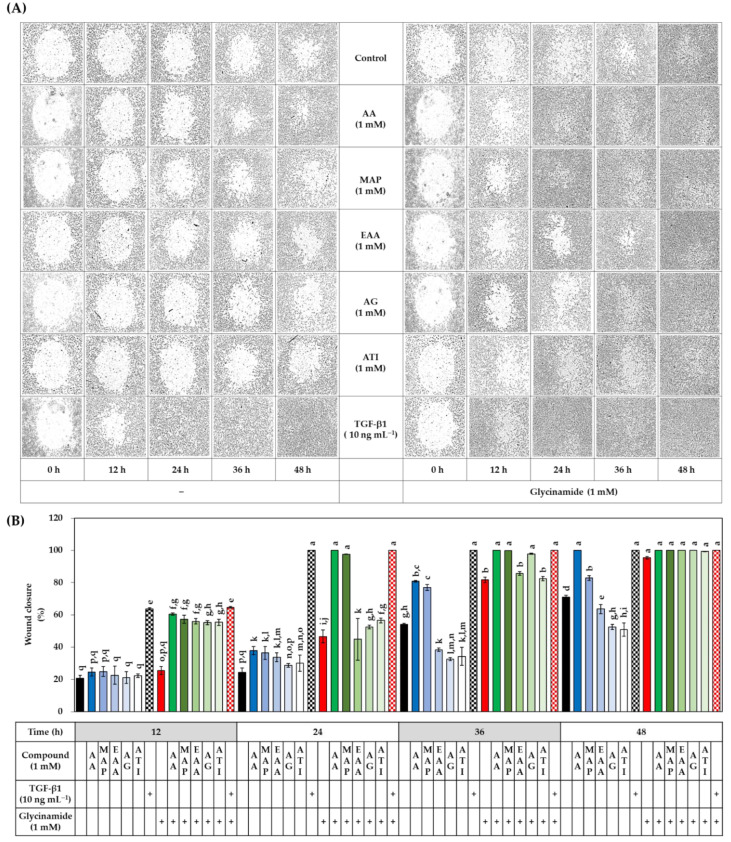
Effects of individual and combined treatments of AA analogs (such as AA, MAP, EAA, AG, and ATI), TGF-β1, and glycinamide on the wound closure in HDFs. Cells were cultured for 12 h in the presence of a stopper to create a small circular wound. After removing the stopper, cells were treated with an AA analog, or TGF-β1 for 48 h in the absence or presence of glycinamide. Representative microscopic wound images before and after treatments are shown in panel (**A**). Quantitated data for wound closure (%) are presented in panel (**B**) (means ± SD, *n* = 3). Groups that share the same alphabet letters (a–q) do not have significantly different means at the *p* < 0.05 level.

The effects of several AA analogs on wound closure were compared in the absence or presence of glycinamide. TGF-β1 was tested for comparative purposes. The cells were treated with vehicle or drugs for 48 h to monitor wound closure over time. As shown in Figure 12, TGF-β1 significantly enhanced wound closure compared to the control group at 12 h and 24 h after treatments. The effects of AA derivatives on wound closure appeared in various patterns. MAP promoted wound closure to a degree similar to that of AA, and the effect was further enhanced by glycinamide. Other derivatives, such as EAA, AG, and ATI, slightly promoted wound closure in the early stages (24 h) but rather inhibited wound closure in the later stages (36 h and 48 h). Glycinamide alone promoted wound closure, and the effect was further enhanced by combined treatment with MAP, but not by combined treatment with EAA, AG, or ATI. These results indicate that each AA analog has a different effect on wound closure, and glycinamide promotes wound closure in HDFs under various conditions in the absence or presence of AA analogs or TGF-β1.

## 4. Discussion

Past studies have focused on proline supplementation to promote collagen production. Perhaps there was a view that proline is an amino acid with a more limited supply than glycine [22,23,24]. However, a recent study reported that the supply of glycine promoted the collagen production in chondrocytes more effectively than the supply of proline or other amino acids [28]. In our current study, as a result of comparing all naturally occurring free amino acids, glycine was verified to enhance collagen production in human dermal fibroblasts most effectively, followed by proline, isoleucine, glutamine, and leucine. This result is very reasonable considering that 1/3 of the amino acids constituting collagen are glycine residues [1]. Thus, it suggested that the supply of glycine is the most important factor for promoting collagen production regardless of the cell type.

This study showed that several kinds of amidated amino acids could enhance the collagen production in HDFs at concentrations that did not affect cell viability. In particular, the collagen production-enhancing effect of glycinamide was the most potent among the amidated amino acids. In addition, its effect was superior to that of glycine and other glycine analogs, such as N-acetyl glycine, N-acetyl glycinamide, glycine methyl ester, glycine ethyl ester, and glycyl glycine. These findings suggest that glycinamide is a very useful form of glycine analog to promote collagen production.

The action of AA is involved not only in the gene transcriptional stage but also in the post-translation modification stage of collagen protein production [18,19,20,21]. The mechanism by which glycinamide promotes collagen production appeared to be different from that of AA. As shown in this study, AA increased the mRNA level of procollagen genes, such as *COL1A1* and *COL3A1*, and the protein level of collagen I. In contrast, glycinamide did not increase the mRNA levels of *COL1A1* and *COL3A1* but increased the protein levels of collagen I and III, and the latter effect was further enhanced by AA. Therefore, glycinamide is assumed to provide a necessary building block at the translation stage of procollagen protein production, without effect at the gene transcription stage. The difference in the mechanisms of action of AA and glycinamide in the process of collagen production will enable a synergistic effect from the combination of these substances.

The function of fibroblasts is very important in wound healing [35]. In the healing process, fibroblasts produce the majority of ECM components, and these ECM molecules, in turn, modulate the function of the fibroblasts [36]. Thus, the interaction of the fibroblast with the ECM is considered a form of autocrine regulation of the wound healing process. In this study, the combination of AA and glycinamide increased not only collagen production but also wound healing as effectively as TGF-β1. TGF-β1 alone was effective in promoting wound healing, and its effect was further enhanced by AA and glycinamide at early time points.

During the proliferative phase of wound healing, fibroblasts can differentiate into contractile myofibroblasts, effectively expressing a specific panel of ECM molecules and inducing wound contraction, contributing to the formation of a temporary skin barrier prior to the remodeling phase [34,37]. Differentiation from fibroblasts to myofibroblasts is mediated by TGF-β1 signaling [38]. In this study, TGF-β1 induced the expression of α-smooth muscle actin, a marker of differentiation from fibroblasts to myofibroblasts, but AA and glycinamide had no such effect. Therefore, it is thought that AA and glycinamide provide biological effects other than the induction of cell differentiation. Myofibroblasts increase in the early stages of dermal wound healing but decrease in the later stages, which is associated with the formation and disappearance of scars [37]. Therefore, if the combination of AA and glycinamide can promote collagen production without inducing cell differentiation, it will provide a new strategy to promote wound healing while minimizing scar formation.

Interestingly, AA and glycinamide in combination increased both collagen I and III, whereas AA alone increased the proportion of collagen I, and glycinamide alone increased the proportion of collagen III. This suggests that the production of collagen I and III is regulated differently. The ratio of collagen III to collagen I is low in aged skin and keloid scars, and high in hypertrophic scars [13,39,40]. If the relative production of collagen I and III can be controlled by changing the combination ratio of AA and glycinamide, it would provide added benefits for the control of skin aging and scarring.

Several types of AA derivatives have been developed that might be more advantageous than AA for topical applications. It was reported that MAP was more stable than AA and enhanced the collagen production in HDFs [41,42]. EAA and AG are used in cosmetics for skin whitening [43,44]. ATI is used in various cosmetic formulations because of its stability and easier skin absorption [45,46]. In our current study, MAP, EAA, AG, and ATI promoted collagen production to a degree similar to that of AA, and when combined with glycinamide, additional collagen production promoting effects were seen. However, in the presence of TGF-β1, the AA derivatives had varying effects on collagen production and cell proliferation. In addition, they had different effects on wound closure. Among the AA derivatives, MAP promoted wound closure similarly to AA, but others were ineffective. Therefore, it is worth noting that there is a significant difference in the biological properties between AA derivatives.

Although HDFs were used as an experimental model in this study, the findings with HDFs would be applied to various other cells, such as lung fibroblasts, endothelial cells, and chondrocytes [25,28]. The combined effect of glycinamide and AA on collagen production and wound healing is assumed to appear in all types of cells. Further studies are needed to address this issue. It would be interesting to examine whether this strategy can prevent skin aging and promote the healing of wounds and the restoration of cartilages, bones, and hairs in vivo.

In addition to being used as a building block in the production of proteins such as collagen, glycine performs various functions essential for human health [47,48]. As a glycine analog, glycinamide is assumed to perform diverse nutritional and pharmacological functions. In certain cases, glycinamide shows very different properties compared to glycine, despite only a minor difference in chemical structure. We have reported that glycinamide, but not glycine, inhibited cellular melanin production stimulated by α-melanocytes stimulating hormone, by acting as an antagonist of melanocortin 1 receptor [49,50,51]. In the current study, the superior effect of glycinamide compared to glycine was demonstrated in enhancing collagen production. Therefore, research on the biological activity of various amino acid derivatives is expected to open a new area for developing biomedicines.

## 5. Conclusions

Among the various amidated amino acids and free amino acids, glycinamide most effectively enhanced the collagen production, and its effect was superior to that of glycine and other glycine analogs, such as N-acetyl glycine, N-acetyl glycinamide, glycine methyl ester, glycine ethyl ester, and glycyl glycine. The collagen production-enhancing effect of glycinamide was further enhanced by combination with AA analogs, such as AA, MAP, EAA, AG, and ATI. The combination of glycinamide and AA or MAP synergistically enhanced wound closure in HDFs to a level similar to that in cells treated with TGF-β1. Therefore, this study presents a potential therapeutic strategy for enhanced collagen production and wound healing. Further in vivo and clinical studies are needed to validate the usefulness of this strategy.

## Figures and Tables

**Table 1:** Single-letter codes for free and amidated amino acids used in this study.

Free Amino Acids	Single-Letter Codes	Amidated Amino Acids	Single-Letter Codes
L-Alanine	A	L-Alaninamide HCl	A-NH_2_
L-Cysteine	C	L-Cysteinamide HCl	C-NH_2_
L-Aspartic acid	D	L-Aspartic acid α-amide HCl	D-NH_2_
L-Glutamic acid	E	L-Glutamic acid α-amide	E-NH_2_
L-Phenylalanine	F	L-Phenylalaninamide HCl	F-NH_2_
Glycine	G	Glycinamide HCl	G-NH_2_
L-Histidine	H	L-Histidinamide HCl	H-NH_2_
L-Isoleucine	I	L-Isoleucinamide HCl	I-NH_2_
L-Lysine	K	L-Lysinamide diHCl	K-NH_2_
L-Leucine	L	L-Leucinamide HCl	L-NH_2_
L-Methionine	M	L-Methioninamide HCl	M-NH_2_
L-Asparagine	N	L-Asparaginamide HCl	N-NH_2_
L-Proline	P	L-Prolinamide HCl	P-NH_2_
L-Glutamine	Q	L-Glutaminamide HCl	Q-NH_2_
L-Arginine	R	L-Argininamide diHCl	R-NH_2_
L-Serine	S	L-Serinamide HCl	S-NH_2_
L-Threonine	T	L-Threoninamide HCl	T-NH_2_
L-Valine	V	L-Valinamide HCl	V-NH_2_
L-Tryptophane	W	L-Tryptophanamide HCl	W-NH_2_
L-Tyrosine	Y	L-Tyrosinamide HCl	Y-NH_2_

**Table 2:** Sequences of primers used for the quantitative reverse transcriptase-polymerase chain reaction (qRT-PCR).

Gene Name	GenBank Accession Number	Forward (F) and Reverse (R) Primer Sequences	References
Collagen I alpha 1 chain (*COL1A1*)	NM_000088.4	F: 5′-GGGATTCCCTGGACCTAAAG-3′ R: 5′-GGAACACCTCGCTCTCCA-3′	[30]
Collagen III alpha 1 chain (*COL3A1*)	NM_000090.4	F: 5′-GGACCTCCTGGTGCTATAGGT-3′ R: 5′-CGGGTCTACCTGATTCTCCAT-3′	[30]
Glyceraldehyde-3-phosphate dehydrogenase (*GAPDH*)	NM_002046.3	F: 5′- ATGGGGAAGGTGAAGGTCG -3′ R: 5′- GGGGTCATTGATGGCAACAA -3′	[31]

## Data Availability

Not applicable.

## References

[1] Ferreira A.M., Gentile P., Chiono V., Ciardelli G. (2012). Collagen for bone tissue regeneration. Acta Biomater..

[2] Samad N.A.B.A., Sikarwar A.S. (2016). Collagen: New Dimension in Cosmetic and Healthcare. Int. J. Biochem. Res. Rev..

[3] Sun B. (2021). The mechanics of fibrillar collagen extracellular matrix. Cell Rep. Phys. Sci..

[4] Shin J.-W., Kwon S.-H., Choi J.-Y., Na J.-I., Huh C.-H., Choi H.-R., Park K.-C. (2019). Molecular Mechanisms of Dermal Aging and Antiaging Approaches. Int. J. Mol. Sci..

[5] Cole M.A., Quan T., Voorhees J.J., Fisher G.J. (2018). Extracellular matrix regulation of fibroblast function: Redefining our perspective on skin aging. J. Cell Commun. Signal..

[6] Sandhu S.V., Gupta S., Bansal H., Singla K., Yadav N.S. (2012). Collagen in Health and Disease. J. Orofac. Res..

[7] Wilkinson H.N., Hardman M.J. (2020). Wound healing: Cellular mechanisms and pathological outcomes. Open Biol..

[8] Li B., Wang J.H.-C. (2011). Fibroblasts and myofibroblasts in wound healing: Force generation and measurement. J. Tissue Viability.

[9] Mathew-Steiner S.S., Roy S., Sen C.K. (2021). Collagen in Wound Healing. Bioengineering.

[10] Tzaphlidou M. (2004). The role of collagen and elastin in aged skin: An image processing approach. Micron.

[11] Quan T., Fisher G.J. (2015). Role of Age-Associated Alterations of the Dermal Extracellular Matrix Microenvironment in Human Skin Aging: A Mini-Review. Gerontology.

[12] Imokawa G., Ishida K. (2015). Biological Mechanisms Underlying the Ultraviolet Radiation-Induced Formation of Skin Wrinkling and Sagging I: Reduced Skin Elasticity, Highly Associated with Enhanced Dermal Elastase Activity, Triggers Wrinkling and Sagging. Int. J. Mol. Sci..

[13] Cheng W., Yan-Hua R., Fang-Gang N., Guo-An Z. (2011). The content and ratio of type I and III collagen in skin differ with age and injury. Afr. J. Biotechnol..

[14] Gelse K., Poschl E., Aigner T. (2003). Collagens—structure, function, and biosynthesis. Adv. Drug Deliv. Rev..

[15] Morikawa M., Derynck R., Miyazono K. (2016). TGF-beta and the TGF-beta Family: Context-Dependent Roles in Cell and Tissue Physiology. Cold Spring Harb. Perspect. Biol..

[16] Phillips C.L., Tajima S., Pinnell S.R. (1992). Ascorbic acid and transforming growth factor-beta 1 increase collagen biosynthesis via different mechanisms: Coordinate regulation of pro alpha 1(I) and Pro alpha 1(III) collagens. Arch. Biochem. Biophys..

[17] Kwak J.Y., Park S., Seok J.K., Liu K.-H., Boo Y.C. (2015). Ascorbyl coumarates as multifunctional cosmeceutical agents that inhibit melanogenesis and enhance collagen synthesis. Arch. Dermatol. Res..

[18] Murad S., Tajima S., Johnson G.R., Sivarajah S.A., Pinnell S.R. (1983). Collagen Synthesis in Cultured Human Skin Fibroblasts: Effect of Ascorbic Acid and Its Analogs. J. Investig. Dermatol..

[19] Maione-Silva L., De Castro E.G., Nascimento T.L., Cintra E.R., Moreira L.C., Cintra B.A.S., Valadares M.C., Lima E.M. (2019). Ascorbic acid encapsulated into negatively charged liposomes exhibits increased skin permeation, retention and enhances collagen synthesis by fibroblasts. Sci. Rep..

[20] Geesin J.C., Darr D., Kaufman R., Murad S., Pinnell S.R. (1988). Ascorbic Acid Specifically Increases Type I and Type III Procollagen Messenger RNA Levels in Human Skin Fibroblasts. J. Investig. Dermatol..

[21] Tajima S., Pinnell S.R. (1996). Ascorbic acid preferentially enhances type I and III collagen gene transcription in human skin fibroblasts. J. Dermatol. Sci..

[22] Karna E., Szoka L., Huynh T.Y.L., Palka J.A. (2020). Proline-dependent regulation of collagen metabolism. Cell. Mol. Life Sci..

[23] Bellon G., Monboisse J., Randoux A., Borel J. (1987). Effects of preformed proline and proline amino acid precursors (including glutamine) on collagen synthesis in human fibroblast cultures. Biochim. Biophys. Acta.

[24] Kay E.J., Koulouras G., Zanivan S. (2021). Regulation of Extracellular Matrix Production in Activated Fibroblasts: Roles of Amino Acid Metabolism in Collagen Synthesis. Front. Oncol..

[25] Krupsky M., Kuang P.-P., Goldstein R.H. (1997). Regulation of Type I Collagen mRNA by Amino Acid Deprivation in Human Lung Fibroblasts. J. Biol. Chem..

[26] Karna E., Miltyk W., Wołczyński S., Pałka J. (2001). The potential mechanism for glutamine-induced collagen biosynthesis in cultured human skin fibroblasts. Comp. Biochem. Physiol. Part B Biochem. Mol. Biol..

[27] Szoka L., Karna E., Hlebowicz-Sarat K., Karaszewski J., Palka J.A. (2017). Exogenous proline stimulates type I collagen and HIF-1α expression and the process is attenuated by glutamine in human skin fibroblasts. Mol. Cell. Biochem..

[28] Lugo P.D.P., Lupiáñez J.A., Meléndez-Hevia E. (2018). High glycine concentration increases collagen synthesis by articular chondrocytes in vitro: Acute glycine deficiency could be an important cause of osteoarthritis. Amino Acids.

[29] Denizot F., Lang R. (1986). Rapid colorimetric assay for cell growth and survival. Modifications to the tetrazolium dye procedure giving improved sensitivity and reliability. J. Immunol. Methods.

[30] Ohto-Fujita E., Konno T., Shimizu M., Ishihara K., Sugitate T., Miyake J., Yoshimura K., Taniwaki K., Sakurai T., Hasebe Y. (2011). Hydrolyzed eggshell membrane immobilized on phosphorylcholine polymer supplies extracellular matrix environment for human dermal fibroblasts. Cell Tissue Res..

[31] Ha J.W., Song H., Hong S.S., Boo Y.C. (2019). Marine Alga Ecklonia cava Extract and Dieckol Attenuate Prostaglandin E2 Production in HaCaT Keratinocytes Exposed to Airborne Particulate Matter. Antioxidants.

[32] An S.-M., Koh J.-S., Boo Y.-C. (2009). Inhibition of melanogenesis by tyrosinase siRNA in human melanocytes. BMB Rep..

[33] Mun G.I., Park S., Kremerskothen J., Boo Y.C. (2014). Expression of synaptopodin in endothelial cells exposed to laminar shear stress and its role in endothelial wound healing. FEBS Lett..

[34] Rodrigues M., Kosaric N., Bonham C.A., Gurtner G.C. (2019). Wound Healing: A Cellular Perspective. Physiol. Rev..

[35] Bainbridge P. (2013). Wound healing and the role of fibroblasts. J. Wound Care.

[36] Tracy L.E., Minasian R.A., Caterson E. (2016). Extracellular Matrix and Dermal Fibroblast Function in the Healing Wound. Adv. Wound Care.

[37] Huang J., Heng S., Zhang W., Liu Y., Xia T., Ji C., Zhang L.J. (2022). Dermal extracellular matrix molecules in skin development, homeostasis, wound regeneration and diseases. Seminars in Cell & Developmental Biology.

[38] Ye Z., Li W., Jiang Z., Wang E., Wang J. (2021). An intermediate state in trans-differentiation with proliferation, metabolic, and epigenetic switching. iScience.

[39] Gauglitz G.G., Korting H.C., Pavicic T., Ruzicka T., Jeschke M.G. (2010). Hypertrophic Scarring and Keloids: Pathomechanisms and Current and Emerging Treatment Strategies. Mol. Med..

[40] Friedman D.W., Boyd C.D., MacKenzie J.W., Norton P., Olson R.M., Deak S.B. (1993). Regulation of Collagen Gene Expression in Keloids and Hypertrophic Scars. J. Surg. Res..

[41] Geesin J.C., Gordon J.S., Berg R.A. (1992). Magnesium Ascorbyl-2-Phosphate Is a More Stable Alternative to Ascorbic-Acid in the Stimulation of Collagen-Synthesis in Human Dermal Fibroblasts. J. Investig. Dermatol..

[42] Smaoui S., Ben Hlima H., Kadri A. (2013). Application of l-Ascorbic Acid and its Derivatives (Sodium Ascorbyl Phosphate and Magnesium Ascorbyl Phosphate) in Topical Cosmetic Formulations: Stability Studies. J. Chem. Soc. Pak..

[43] Liao W.C., Huang Y.-T., Lu L.-P., Huang W.-Y. (2018). Antioxidant Ability and Stability Studies of 3-O-Ethyl Ascorbic Acid, a Cosmetic Tyrosinase Inhibitor. J. Cosmet. Sci..

[44] Huang S.-C., Lin C.-C., Wen K.-C. (2004). Simultaneous determination of magnesium ascorbyl phosphate, ascorbyl glucoside, kojic acid, arbutin and hydroquinone in skin whitening cosmetics by HPLC. J. Food Drug Anal..

[45] Machado N., dos Santos L., Carvalho B., Singh P., Soto C.T., Azoia N., Cavaco-Paulo A., Martin A., Favero P. (2016). Assessment of penetration of Ascorbyl Tetraisopalmitate into biological membranes by molecular dynamics. Comput. Biol. Med..

[46] de Almeida M.M., Lima C.R.R.D., Quenca-Guillen J.S., Moscardini E., Mercuri L.P., Santoro M.I.R.M., Kedor-Hackmann E.R.M. (2010). Stability evaluation of tocopheryl acetate and ascorbyl tetraisopalmitate in isolation and incorporated in cosmetic formulations using thermal analysis. Braz. J. Pharm. Sci..

[47] Koopman R., Caldow M.K., Ham D.J., Lynch G.S. (2017). Glycine metabolism in skeletal muscle: Implications for metabolic homeostasis. Curr. Opin. Clin. Nutr. Metab. Care.

[48] Wang W., Wu Z., Dai Z., Yang Y., Wang J., Wu G. (2013). Glycine metabolism in animals and humans: Implications for nutrition and health. Amino Acids.

[49] Kim J.H., Seok J.K., Kim Y.M., Boo Y.C. (2019). Identification of small peptides and glycinamide that inhibit melanin synthesis using a positional scanning synthetic peptide combinatorial library. Br. J. Dermatol..

[50] Boo Y.C. (2020). Up- or Downregulation of Melanin Synthesis Using Amino Acids, Peptides, and Their Analogs. Biomedicines.

[51] Boo Y., Jo D., Oh C., Lee S., Kim Y. (2020). The First Human Clinical Trial on the Skin Depigmentation Efficacy of Glycinamide Hydrochloride. Biomedicines.

